# Successful live birth of thin endometrium: A case report

**DOI:** 10.1097/MD.0000000000037399

**Published:** 2024-03-01

**Authors:** Baoyi Huang, Danying Lu, Yanxiang Kong, Lin Ma

**Affiliations:** a The Reproductive Medical Center, The Seventh Affiliated Hospital, Sun Yat-sen University, Shenzhen, Guangdong, China.

**Keywords:** endometrial thickness, infertility, spontaneous pregnancy

## Abstract

**Rationale:**

The success of pregnancy depends on various factors, with the endometrial receptivity being a crucial component. Endometrial thickness (EMT) serves as a direct indicator for assessing endometrial receptivity. Previous studies have suggested that a thin endometrium is associated with lower pregnancy rates, especially in patients with an EMT of less than 4 mm. Even in assisted reproductive technology cycles with high success rates, clinical pregnancy cases in patients with such thin endometrium are reported to be very few, let alone in natural conception cycles. Therefore, a thin endometrium poses significant challenges for infertility patients. In this study, patients with an extremely thin endometrium were able to achieve clinical pregnancy and successful live births through natural conception, highlighting the possibility of success even in challenging cases.

**Patient concerns:**

The patient presented with polycystic ovary syndrome and ovulation disorders. She underwent a natural cycle of letrozole-induced ovulation. On the day of the human chorionic gonadotropin trigger, she had an EMT of 3.8 mm.

**Diagnoses:**

Polycystic ovary syndrome, ovulation disorders, thin endometrium.

**Interventions:**

The patient received medications including Progynova, Aspirin, and Dydrogesterone.

**Outcomes:**

The patient achieved spontaneous conception and subsequently had a live birth.

**Lessons:**

This case report underscores the significance of managing a thin endometrium during letrozole-induced ovulation. While EMT is traditionally pivotal for predicting embryo implantation success, our findings indicate that endometrial receptivity extends beyond thickness alone. Factors such as endometrial morphology, type, and blood supply play crucial roles. Successful pregnancies with a 3.8 mm EMT are rare, making this case a beacon of hope for such patients. It highlights that, with appropriate interventions, successful pregnancies remain attainable. For those with a thin endometrium, emphasis should extend beyond thickness, addressing ways to enhance both endometrial blood supply and morphology for improved pregnancy rates.

## 1. Introduction

Synchrony between early embryo development and receptive endometrium is necessary for implantation and subsequent pregnancy progression.^[1]^ Poor endometrial receptivity and altered embryo-endometrial dialogue have been suggested to be responsible for two-thirds of implantation failures.^[2]^ At present, there are many commonly used methods to evaluate endometrial receptivity in clinical practice. Ultrasonography has become one of the commonly used methods to evaluate endometrial receptivity in clinical practice due to its simple operation and noninvasive advantages. Parameters assessed by ultrasound for endometrial receptivity mainly included endometrial thickness (EMT), endometrial pattern, and endometrial and subendometrial blood flow.^[3,4]^

There is currently no clear definition of thin endometrium. Some studies have suggested that embryo implantation and live birth rates were higher when the endometrium was larger than 8 mm on human chorionic gonadotropin (hCG) day^[5]^ while some studies suggested that the cutoff value was 7 mm,^[6]^ and even 6 mm.^[7]^

The thinnest EMT currently reported to be capable of a healthy full-term live birth in assisted conception is 3.5 mm.^[8]^ We present a case of female ovulatory dysfunction, where a thin endometrium, with an EMT of <4 mm, was observed following letrozole-induced ovulation. However, after undergoing drug treatment, the patient successfully achieved spontaneous pregnancy and live birth.

## 2. Case presentation

The patient has given consent to share their diagnosis, treatment process, test results, examinations, and pregnancy outcomes in the case report, and has obtained the patient’s written informed consent for publication

### 2.1. Basic information

The patient reported in this case was a 25-year-old anovulatory female with fertility needs. The patient had irregular menstruation, menstrual cycle of 1 to 6 months and oligomenorrhea. The patient visited previous hospital due to irregular menstruation in 2020, and was diagnosed with “polycystic ovary syndrome and insulin resistance.” She was treated with oral metformin and intermittent use of Diane-35 for menstruation regulation. In 2021 the patient was married and experienced spontaneous abortion at 7 weeks of gestation without uterine curettage in December. Before seeking medical attention, the patient had already taken oral contraceptives to regulate her menstrual cycle for 6 months. Due to ovulatory dysfunction, on the second day of menstruation, the patient visited hospital for ovulation induction to assist pregnancy.

### 2.2. Physical examination

The patient’s height was 155 cm, weight was 59.5 kg, Body Mass Index was 24.77kg/m^2^, Except for increased hair in the extremities, axillae, and vulva, there were no other clinical manifestations of hyperandrogenism. Gynecological examination showed no significant abnormal findings.

### 2.3. Laboratory, imaging examination or other tests

Anti-Mullerian hormone (AMH) showed 4.03 ng/mL. Sex hormones indicated elevated total testosterone levels, follicle-stimulating hormone/luteinizing hormone (LH): 6.29/10.32. Gynecological ultrasound indicates polycystic changes in both ovaries, with no abnormalities remaining. Prepregnancy eugenic examination was generally normal. The semen test results of the male partner showed normal.

### 2.4. Treatment

According to the Rotterdam diagnostic criteria for polycystic ovary syndrome (PCOS),^[9]^ the patient exhibited irregular menstrual cycles, bilateral polycystic ovaries changes, and laboratory evidence of hyperandrogenism. After excluding other conditions causing elevated androgen levels, a diagnosis of polycystic ovary syndrome was established. The patient displayed PCOS-related features, including elevated levels of AMH and LH in sex hormones. PCOS can lead to female infertility through ovulatory dysfunction.

**Figure 1.**
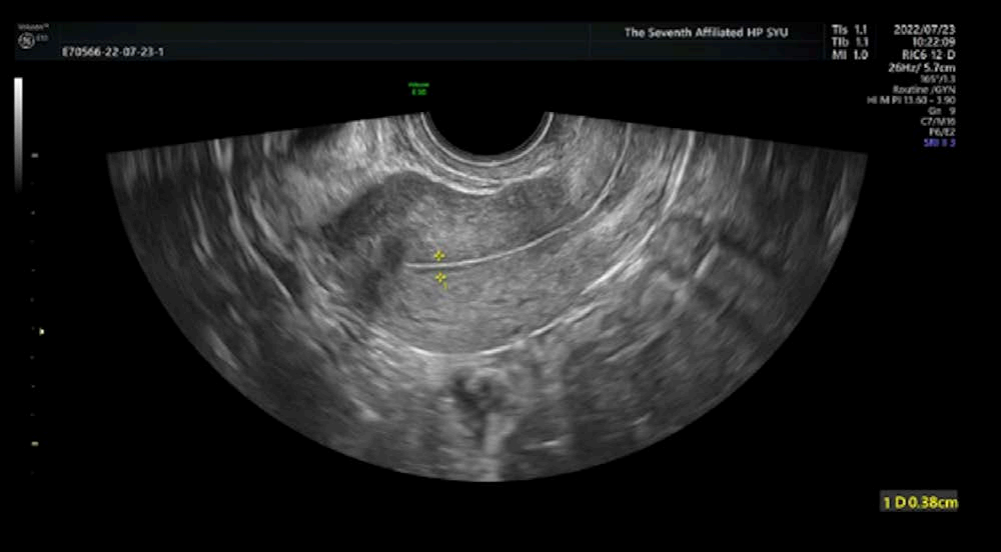
Endometrial ultrasound image on hCG day. On the day of HCG trigger, the thickness of the endometrium is 3.8 mm, and the echo of the endometrium is uniform, presenting a “three line sign”, which is consistent with the appearance of A-type endometrium.

Because of ovulatory dysfunction, the patient had a need for ovulation induction to assist pregnancy. After excluding contraindications to ovulation induction, oral letrozole was started on the fifth day of menstruation for ovulation induction, starting dose was 2.5 mg orally for 5 days. Ultrasound monitoring on the 10th day of menstruation revealed a dominant follicle 11*9 mm, and EMT (2.9 mm) grew slightly lagged behind the follicular development. On the 12th day of menstruation, follicular development was more asynchronous with endometrial development, reaching 16*13 mm in follicles and only 4.1 mm in endometrium. estradiol valerate (Progynova, 1 mg bis in die, in Latin, twice a day [bid] po) were given to promote endometrial growth. On the 14th day of menstruation, ultrasound showed follicles grew to 19*15 mm, EMT was 3.8 mm, further follicular development, but the responsiveness of endometrium to Progynova was poor, so the dose of Progynova was adjusted to 1 mg tid, and Aspirin Enteric-coated (100 mg quaque die, once a day [qd] po) was added to prevent thrombosis while it was expected that aspirin could improve endometrial blood supply and increased the responsiveness of endometrium to Progynova. On the 15th day of menstruation, the matured follicles grew to 24*19 mm and EMT was 3.8 mm (Fig. 1) and the endometrium showed a 3-line sign. The appearance of LH surge suggested follicular maturation and hCG trigger was prepared to rupture follicle.

The patient was explained about the condition during follicular monitoring: the endometrium was not synchronized with follicular development, and due to the thin endometrium, it may affect subsequent embryo implantation and conception. Even if the condition of the endometrium was not ideal, the patient still insisted on trying to conceive, so she was guided to have sexual intercourse while the hCG trigger ruptured follicle. Two days later, the patient returned for ultrasound which revealed ovulation, when the EMT was 4.7 mm. After ovulation, patients were instructed to use Progynova (1 mg tid for 4 days; 1 mg bid for 7 days; 1 mg qd for 3 days) to stimulate endometrial growth and dydrogesterone (10 mg bid for 14 days) for luteal support and endometrial conversion. Aspirin was continued at original dose.

### 2.5. Prognosis and follow-up

On the 28th day of menstruation, the patient had a positive blood hCG test. Because the patient had repeated brown vaginal discharge and abdominal pain, the patient was given oral Duphaston 10 mg tid, vaginal Utrogestan 200 mg qn and intramuscular progesterone injection 20 mg qd for luteal support. Due to vaginal bleeding and pregnancy the patient was instructed to stop aspirin. On the 45th day of menstruation, gynecologic ultrasound showed clinical pregnancy. Gestational hypertension was found by prenatal examination at 30 weeks of gestation and a male baby was delivered by cesarean section due to “premature rupture of membranes, fetal distress and gestational hypertension,” at 36^+3^ weeks gestation, with a birth weight of 2.35 kg.

## 3. Discussion

This patient sought medical assistance primarily for fertility purposes. Due to a prior diagnosis at an external institution, the diagnostic basis was not reiterated. Women with PCOS often face ovulatory challenges, necessitating the use of ovulation-inducing medications to facilitate follicular development and ovulation for improved chances of conception. Physical examination revealed the patient’s issues primarily related to being overweight (according to Chinese standards) and clinical manifestations of hyperandrogenism.

Laboratory results showed elevated levels of AMH, indicating heightened ovarian reserve and a tendency toward PCOS. Elevated LH levels, increased testosterone levels, and bilaterally polycystic ovaries on imaging aligned with laboratory manifestations of PCOS. Identifying the patient’s ovulatory dysfunction, we opted for letrozole to induce ovulation in this woman with fertility aspirations.

Letrozole, as an aromatase inhibitor, mainly inhibit the activity of aromatase, block the conversion of testosterone to estradiol and cause estrogen relatively insufficient in the body, thereby enhancing the negative feedback effect of estrogen on the hypothalamus-pituitary gland, resulting in increased gonadotropin secretion to achieve the purpose of promoting follicular development.^[10]^ Because estrogen levels in the body decline relatively, estrogen support of the endometrium is insufficient, therefore, thin endometrium may appear in letrozole ovulation induction cycles, endometrial development cannot keep up with follicular development. Correction should be achieved by adding estrogen promptly. Compared with clomiphene citrate, most studies suggested that letrozole had little effect on the endometrium,^[11,12]^ but in clinical practice, there were many patients similar to this case. Despite the dominance of follicular development in this patient, there was a significant lag in endometrial development compared to follicular growth. In other words, a mismatch between dominant follicular development and endometrial development was observed. This theoretical discrepancy could severely impact the patient’s chances of conception. Consequently, upon identifying this situation, we employed the commonly used medication estradiol valerate (Progynova) to promote endometrial growth and development. However, even after multiple escalations in the dosage of estradiol valerate, the patient’s EMT remained at a level highly unfavorable for conception, leading to frustration for both the clinical team and the patient.

Current studies have reported that EMT on the day of hCG trigger had an impact on pregnancy,^[13]^ and its thin endometrium was a clinical challenge, especially in patients with chemoradiotherapy or severe intrauterine adhesions. In response to the problem of thin endometrium, many strategies to promote endometrial growth have been adopted in clinical practice, including estrogen administration, low-dose aspirin,^[14]^ vitamin E,^[15]^ vaginal sildenafil citrate,^[15]^ and Intrauterine perfusion with granulocyte colony-stimulating factor.^[16]^ Successful pregnancy after intrauterine instillation of autologous bone marrow stem cells combined with platelet-rich plasma has also been reported.^[16]^There are also case reports of increased thin endometrial receptivity and live birth through the use of autologous endometrial mesenchymal stromal/stem cells in 2020.^[17]^ In the present case, only estrogen, aspirin, vitamin E were used for endometrial stimulation because the patient had no obvious organic endometrial lesions.

Aspirin is a nonsteroidal anti-inflammatory drug, which is clinically mainly used to antipyretic, anti-inflammatory, and antiplatelet aggregation. Aspirin can promote endometrial microvessel formation, improve local blood circulation, and increase EMT.^[18]^ Aspirin may inhibit endometrial fibrosis and improve endometrial receptivity in patients with intrauterine adhesions by inhibiting the transforming growth factor-β1-Smad2/Smad3 pathway.^[19]^ In 2022, a study reported that the use of low-dose aspirin in patients with unexplained recurrent implantation failure could improve the pulsatility index, resistive index and systolic-to-diastolic ratio values of endometrial blood flow, especially the resistance of endometrial and uterine artery blood flow was significantly lower than that before treatment, suggesting that low-dose aspirin therapy could improve endometrial receptivity.^[14]^

For this patient, although the EMT did not increase significantly after the use of aspirin, the patient had a history of embryo arrest, and the endometrium may have endometrial fibrosis or endometrial inflammation. Aspirin not only increased the endometrial blood supply, but also eliminated the inflammatory response and inhibited endometrial fibrosis, improving the receptivity of the endometrium to the embryo, which may also be the key reason why the patient still successfully conceived under the condition of thin endometrium.

One study concluded that endometrial blood flow was better predictive of endometrial receptivity than EMT and endometrial pattern, and endometrial blood flow in Doppler was positively correlated with pregnancy outcome.^[20]^ The shortcoming of this study is that no endometrial blood flow analysis was performed, endometrial blood flow could not be assessed on the day of hCG trigger, and it could not confirm whether endometrial blood supply was improved after oral aspirin administration.

This study has potential limitations. Firstly, as a retrospective case analysis, ultrasound examinations were not consistently performed by the same sonographer, introducing possible measurement variations and errors in EMT data. However, despite this limitation, the ultrasound images still clearly reflected the EMT and morphology. Secondly, although the patient had a history of miscarriage, the absence of dilation and curettage makes the likelihood of endometrial damage low. The thin endometrium observed in this patient is primarily attributed to the inhibitory effect of letrozole on estrogen, rather than endometrial damage. Additionally, the patient indirectly excluded the impact of solely EMT on clinical pregnancy outcomes, focusing instead on endometrial morphology and blood supply. Previous research has highlighted the brief window of endometrial receptivity, requiring coordination between endometrial status and embryo implantation.^[21]^ Despite the patient having an EMT below 4mm on the day of ovulation and a poor response to drugs promoting endometrial growth, successful pregnancy occurred under favorable conditions of good endometrial morphology and blood supply. Lastly, due to the constraints of a retrospective case analysis, other ultrasound indicators influencing endometrial receptivity, such as uterine artery blood flow, endometrial elastography, mid-luteal phase EMT, and blood flow signals, were not analyzed. These factors are potential influences that should be considered for further investigation in future studies.

## 4. Conclusions

The induction of ovulation with letrozole has an impact on EMT, and its primary mechanism may be related to the decrease in estrogen levels after aromatase inhibition. It is crucial to be vigilant about the lag in endometrial development post letrozole-induced ovulation. Timely detection of endometrial issues through ultrasound monitoring, prompt estrogen supplementation, and stimulation of endometrial proliferation are essential in addressing this concern.

Furthermore, this case report indicates that endometrial receptivity is not solely confined to EMT. Instead, the focus should be on improving factors such as endometrial morphology, type, and enhancing endometrial blood supply. Currently, there are limited reports of successful pregnancies with EMT less than 4mm. This case serves as a beacon of hope for patients with thin endometrium, offering new perspectives and insights for future research.

## Author contributions

**Conceptualization:** Baoyi Huang, Lin Ma.

**Data curation:** Danying Lu, Yanxiang Kong.

**Writing – original draft:** Baoyi Huang, Danying Lu.

**Writing – review & editing:** Baoyi Huang, Lin Ma.
